# Using Automated Point Dendrometers to Analyze Tropical Treeline Stem Growth at Nevado de Colima, Mexico

**DOI:** 10.3390/s100605827

**Published:** 2010-06-09

**Authors:** Franco Biondi, Peter Hartsough

**Affiliations:** 1 DendroLab, Mail Stop 154, University of Nevada, Reno, NV 89557, USA; 2 Graduate Program of Hydrologic Sciences, University of Nevada, Reno, NV 89557, USA

**Keywords:** point dendrometers, radial growth, tree rings, dendroecology, high elevation ecosystems, Mexican mountain pine, *Pinus hartwegii* Lindl., Volcán de Fuego

## Abstract

The relationship between wood growth and environmental variability at the tropical treeline of North America was investigated using automated, solar-powered sensors (a meteorological station and two dendrometer clusters) installed on Nevado de Colima, Mexico (19° 35′ N, 103° 37′ W, 3,760 m a.s.l.). Pure stands of *Pinus hartwegii* Lindl. (Mexican mountain pine) were targeted because of their suitability for tree-ring analysis in low-latitude, high-elevation, North American Monsoon environments. Stem size and hydroclimatic variables recorded at half-hour intervals were summarized on a daily timescale. Power outages, insect outbreaks, and sensor failures limited the analysis to non-consecutive months during 2001–2003 at one dendrometer site, and during 2002–2005 at the other. Combined data from the two sites showed that maximum radial growth rates occur in late spring (May), as soil temperature increases, and incoming short-wave radiation reaches its highest values. Early season (April–May) radial increment correlated directly with temperature, especially of the soil, and with solar radiation. Stem expansion at the start of the summer monsoon (June–July) was mostly influenced by moisture, and revealed a drought signal, while late season relationships were more varied.

## 1. Introduction

Ecological studies provide the necessary background knowledge required to properly interpret a number of paleoclimatic records derived from biological organisms [1,2]. Mountainous and ecotonal regions have been identified as critical zones for understanding eco-hydro-climatic changes over a variety of timescales [3], but dendrochronological records from high latitude and/or high elevation climatic treelines [4] have been the subject of heated controversy regarding their significance for defining human impacts on global surface air temperature [5]. A recent review of climate warming impacts on timberline carbon and water balances in the central European Alps suggested that treeline dynamics respond more to climate extremes than gradual temperature changes [6]. In the western United States, ring-width of bristlecone pine (*Pinus longaeva* D.K. Bailey) growing within about 150 m of the upper treeline limit have reached unprecedented peaks in the last few decades [7]. This trend is matched by increased air temperature in PRISM data [8], although cause-effect mechanisms would have been easier to identify if *in situ* hydroclimatic measurements had been available.

While several investigations have focused on environmental controls of wood growth at mid- or high-latitude treelines e.g., [9,10], relatively fewer studies have focused on low-latitude locations e.g., [11,12]. On the other hand, measurements of tropical forest plots at elevations below treeline have been used to assess long-term changes in forest biomass and carbon cycling [13], and stem size changes have provided information on ecological pathways linked to water cycling in these regions [14–16]. Despite the difficulty of separating radial growth from hydration status of tropical trees [17–19], intensive monitoring of stem dimension can generate data on soil water availability in seasonally dry tropical environments [20]. Baker *et al.* [15] found that shade-tolerant woody species in a Ghana forest displayed little diurnal variation in stem size related to water exchanges, and attributed this to low elastic storage in the trunks. Worbes [21] used stem size measured at high resolution in a Venezuela forest to define the actual growth of tropical trees that either do not have a dormant season or have a very short one, and to reveal differences between evergreen and deciduous tropical species, since the former maintain their stem size, while the latter decrease substantially, throughout the dry season.

Various authors have identified distinct components in the diel cycle of stem size. Herzog *et al.* [22] divided daily fluctuations of Norway spruce stems into five phases, representing rates of change and hydration states of the phloem and xylem within the general framework of nocturnal recharge and daily dehydration. They also suggested that there may be several separate reservoirs of water within the tree that get depleted on various timescales, some diurnal and some longer. This topic has been further investigated through modeling of water flow and stem storage dynamics [23,24]. Dendrometers have been used under both controlled conditions [23,25,26] and in field observational studies [22,27–30] to separate wood radial growth from transient changes caused by water balance components.

The diel stem size cycle is now commonly divided in three distinct phases: a contraction, usually associated with stem desiccation during the day, followed by an expansion, normally in the evening, and a true stem radius increase. This approach was proposed by Downes *et al.* [28] as a simplification of the five-phase approach. The irreversible portion of the diurnal change is the Stem Radial Increment (SRI), which is calculated by comparing the daily maximum of stem size to the previous daily maximum, and is assumed to be zero when the current-day maximum does not reach the previous-day maximum. In order to investigate environmental controls on radial growth of tropical treeline trees, we conducted a multi-year observational field study to quantify the relationship between SRI of *Pinus hartwegii* Lindl. and several hydroclimatic variables controlled by the North American Monsoon System [31,32]. Our main objective was to test the relationship between average ring-width indices at the site and June precipitation in Colima, Mexico, that had been identified in a previous study [11], given that ecological studies provide the necessary information to properly interpret proxy records, such as those derived from tree rings, used in paleoclimatic reconstructions.

## 2. Experimental Section

**Figure 1.**
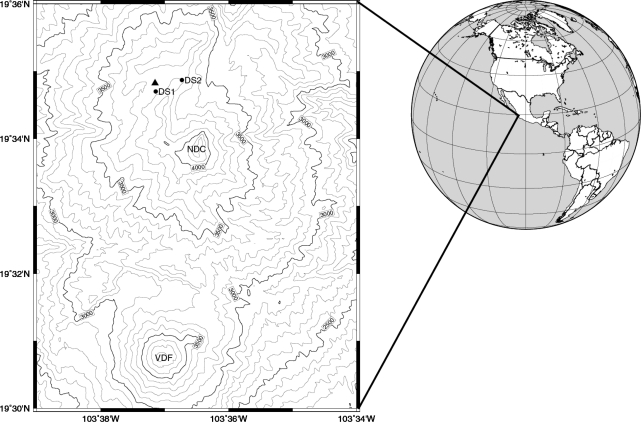
Global and topographic maps showing the location of the study area on Nevado de Colima (NDC), Mexico, in relation to the nearby Volcán de Fuego (VDF). Contour lines are drawn at 100 m intervals; the automated weather station (▴) and two dendrometer sites (•, DS1 and DS2) are about 300 m below treeline [41].

The study site is located on Nevado de Colima, Mexico (Figure 1), in pure, uneven-aged stands of Mexican mountain pine (*Pinus hartwegii* Lindl), the dominant treeline species in tropical North America [33,34]. Nevado is at the western end of the trans-Mexican volcanic belt, which includes several of the tallest mountains in central America [35]. The climate of the study area is typical of the North American Monsoon System [36], with a distinct summer wet season (June–October) and a prolonged dry season (November–May). The dry season is accompanied by cold fronts, occasionally producing limited snow fall at the highest elevations [37].

An automated weather station installed on an open ridge (19°34.778′ N, 103°37.180′ W, 3,760 m a.s.l.) has measured several environmental parameters at half-hour intervals since May 2001 [38]. We used these data to calculate daily summaries of total precipitation (mm), air temperature (maximum and minimum; °C), soil temperature (maximum and minimum; °C), mean soil moisture content (%), mean air relative humidity (%), mean barometric pressure (hPa), total incoming solar radiation (MJ m^−2^), mean wind speed (km hr^−1^) and maximum wind gust (km hr^−1^). Climate regime during the year was then summarized using monthly values. Vapor pressure deficit (hPa) was calculated from half-hourly measurements as the difference between saturation vapor pressure and ambient vapor pressure. The former was computed from mean air temperature (*AT*, °C), as follows (formula derived from Equation 6 in [39]):

(1) $$\mathrm{SVP}=6.11\;e^{\frac{17.2693882\;\mathrm{AT}}{\mathrm{AT}\;+\;237.3}}$$

and the latter was quantified as a function of *SVP* (hPa), mean relative humidity (*RH*, %), and barometric pressure (*P*, hPa) as follows (formula derived from the equations on p. 37 of [40]):

(2) $$\mathrm{VP}=\frac{\mathrm{RH}\;\times \;\mathrm{SVP}\times \;P}{\mathrm{RH}\;\times \mathrm{SVP}\;\;+\;\;100\;\left(P-\mathrm{SVP}\right)}$$

Half-hourly vapor pressure deficit (*SVP*–*VP*) was then summarized by the daily mean.

Dendrometer sensors manufactured by Agricultural Electronics Corp., Tucson, Arizona, USA (http://www.phytogram.com/) were installed at two sites within a one-km radius from the weather station, as explained in detail by Biondi *et al.* [41]. In that study it was found that data from band dendrometers were mostly reflecting bark hydration status and air temperature changes, rather than actual growth. Similarly, point dendrometers installed on the outside of the bark had a low signal-to-noise ratio, with signal being wood formation. Hence, only data from point dendrometers that were installed after shaving off most of the bark are reported in this article. Daily tree growth was quantified by the Stem Radial Increment (SRI), calculated by subtracting the maximum stem size (μm) for the previous day from the maximum stem size (μm) for the current day; negative differences were set to zero, and those >350 μm were considered spurious and set to a missing value. This decision was motivated by the fact that only a handful of such measures existed, while average daily SRI was about 15 μm with standard deviation less than 20 μm. SRI was then compared to daily environmental variables measured by the automated weather station. Cumulative SRI was used to highlight the length of the growing season, and also to compute the percentage of annual growth (= total SRI from January 1st to December 31st) by month. Although air and soil temperature sensors included in the dendrometer packages were installed at both sites, their records were highly discontinuous, and could only be used for spot-checking the agreement with data recorded by the weather station.

Daily values of SRI (at Site 1 and Site 2) were compared to the hydroclimatic variables measured by the automated weather station using the daily summaries mentioned above. Hence, explanatory variables consisted of total precipitation and incoming solar radiation; maximum air, soil temperature, and wind gust; minimum air and soil temperature; mean soil moisture content, air relative humidity, barometric pressure, wind speed, and vapor pressure deficit. Because degrees of freedom are reduced by time-series autocorrelation [42], and many coefficients are computed together, hence requiring adjusted confidence levels [43], bootstrap techniques [44,45] were used to estimate and test correlation coefficients. In addition, given that environmental variables can be mutually correlated, we also included them as predictors in a multivariate analysis, and used principal component regression to correct for multicollinearity [46,47]. Principal components were selected according to the PVP criterion [48], but since principal components with the lowest amount of information are omitted, normal significance levels of regression coefficients can be misleading [46]. Therefore, for each month with at least 21 days of available observations, 1,000 random samples were drawn with replacement from the original daily data, then simple correlations and principal component regression were performed on each of those 1,000 bootstrap samples, and the 50th percentile of correlation or regression coefficients (*i.e.*, the median estimate) was tested for significance by comparing its absolute value to half the difference between the 97.5th and the 2.5th percentile of its 1,000 estimates [49].

## 3. Results

**Figure 2.**
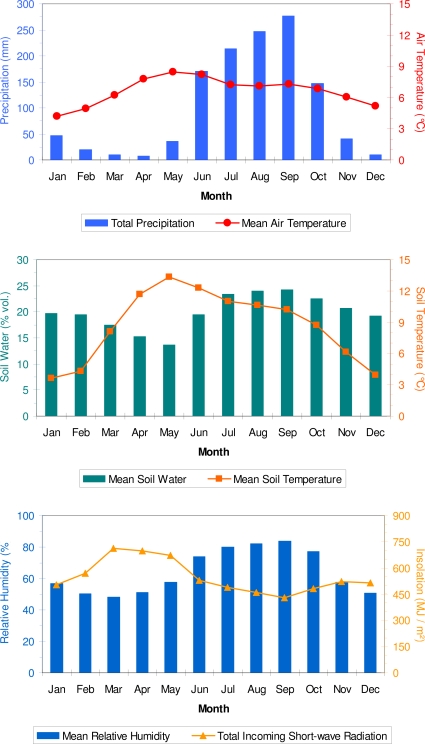
Climatic diagrams showing average monthly variables based on 2002–2008 data from the automated weather station. Soil data were collected at about 30 cm below ground, precipitation at about 1 m above ground, air temperature and relative humidity at about 2 m above ground, and total insolation at about 3 m above ground.

Climatic regime at the study area is characterized by both abundant precipitation and reduced air temperature during the summer monsoon, from June to October (Figure 2). Annual rainfall ranged from 971 mm in 2005 to 1,771 in 2006, with a mean of 1,220 mm during 2002–2008. April had the lowest mean total precipitation (8 mm), and September had the highest one (277 mm). Mean monthly insolation (= incoming solar radiation) was highest in March and lowest in September, as a consequence of the more abundant precipitation (hence cloud cover) in that month (Figure 2). On average, about 85% of the annual precipitation fell from June to October, with a minimum of 73% in 2004 and a maximum of 95% in 2008. Average annual air temperatures were 6.6 °C (mean), 11.5 °C (maximum), and 1.8 °C (minimum); the mean monthly minimum temperature dipped below freezing from December to February. As expected, soil temperatures had a lower annual range, with average values of 8.7 °C (mean), 9.6 °C (maximum), and 7.7 °C (minimum). Mean monthly soil temperature followed the same annual cycle as air temperature, but summer values were above, and winter ones were below, those of mean air temperature (Figure 2). Even in winter (December–February) mean monthly soil temperature was greater than 3.5 °C.

**Figure 3.**
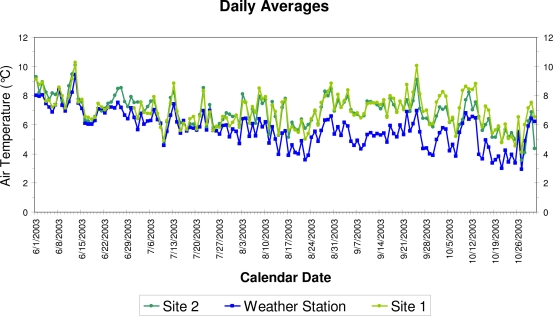
Time series graph of mean daily air temperature measured at the two dendrometer sites, showing good agreement between them, and also with values recorded by the automated weather station (see [41] for details on site features).

The warmest month was May (average air temperature of 8.4 °C), mostly because of high maximum temperature (13.8 °C): its average minimum temperature (3.1 °C) was lower than that of June–September. January was the coldest month (average air temperature of 4.2 °C), both for mean maximum (9.3 °C) and minimum (−0.9 °C) temperature. Air and soil temperature were also measured at the dendrometer sites, but the period of overlap between the two sites and the weather station was limited to June–October 2003 (Figure 3). During this period, linear correlation between mean daily air temperature at the two dendrometer sites was 0.88, and the correlations with the weather station data were 0.77 (Site 1) and 0.80 (Site 2). Average daily air temperature was not significantly different between the two sites (9.13 *vs.* 9.29 °C, p-value = 0.395 using a two-sample t-test), but it was significantly lower at the weather station site (8.00 °C, p-value < 0.001 using a two-sample t-test).

Soil moisture (volumetric water content) at 30 cm depth had an annual average of about 20%, and ranged from a mean of 14% for May, the driest month for tree growth, to a mean of 24% for August and September, the wettest ones (Figure 2). Relative humidity had an annual average of 64%, with monthly means that varied between 48% (March) and 58% (May and November) outside of the monsoon season, but rapidly increasing to 74% in June, and reaching a peak of 84% in September (Figure 2). Monthly insolation was highest from March through May, and then quickly decreased because of the monsoonal clouds, reaching a minimum in September (Figure 2). No overall trend was observed in environmental variables during the period of overlap with dendrometer data (2001–2005), although maximum air temperature has begun increasing in the most recent years, most likely because of reduced wind speed around the time of solar noon [38].

In spring 2003, four of the seven instrumented trees at Site 1 were dying from a pest infestation. At some point during the previous months, round-headed pine beetle (*Dendroctonus adjunctus* Blandford) had spread to several stands in the area. The timing of initial infestation for documented outbreaks in the southwestern USA is usually the fall, late September to early November [50–52], but in this case we documented a decrease in stem size of the infested trees starting in the previous summer (see Figure 5 in [41]). In addition to the insect outbreak, Site 1 also experienced power outages, and at least two were caused by atmospheric electrical activity (including lightning). Site 1 was dismantled in fall 2003 due to logging of the instrumented trees by the Nevado Park managers as part of their beetle treatment program.

Site 2 was located in a more shaded (northern exposure), steeper, and rockier area than Site 1 [41]. In addition to lightning-caused damage, mostly during the summer monsoon, power outages occurred in winter, when a nearby rock outcrop blocked sunshine during much of the day. Hence, Site 2 became fully operational in April 2002, several months after Site 1, and shut down again in June of that year (see Figure 4 in [41]). Dendrometer dataloggers at Site 2 were replaced with an aboveground system and completely rewired in spring 2003. At the same time, a circle of lightning rods was installed around the site, resulting in a nearly continuous record during 2004 and 2005. Due the earlier removal of Site 1, the two sites had a brief period of overlap during 2002 and 2003, which, coupled with the discontinuous nature of the records, hampered cross-site comparisons.

Half-hourly stem size measurements (Figure 4a) showed a daily cycle characterized by a maximum around mid-day, following the stem recharge by soil water during the night and the photosynthesis during the morning, *i.e.*, the expansion phase. This peak was followed by stem shrinking (contraction phase) as air temperature and evapotranspiration increased, although the afternoon and evening were also characterized by relatively large oscillations in stem size. While this pattern was present in all months (Figure 4a), there was a clear difference between the middle of the growing season (early July), when the expansion phase included a wood growth component, and either before (early March) or after (mid-late October) the growing season, when there was little difference in maximum stem size from one day to the next. Cumulative SRI (Figure 4b) represented actual wood growth, as the diurnal swelling and shrinking were removed by computing the daily SRI. During every day of the year (even in winter) cumulative water adsorption by the stem may produce positive SRI values, so that the length of the growing season is best determined using multiple years of dendrometer data (e.g., compare Figure 4 to Figure 5 in [41]).

**Figure 5.**
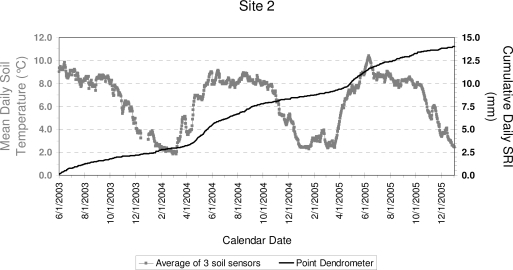
Stem radial size (cumulative daily SRI, black) at Site 2 from June 1st, 2003 to January 1st, 2005. Maximum growth rates occurred in the late spring, when soil temperature measured at the site (gray) increased above 4–6 °C.

**Figure 6.**
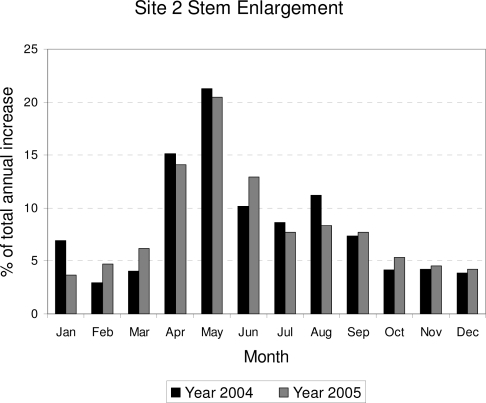
Summary of monthly stem growth rates over two consecutive years at Site 2 (2004–2005). Although stem enlargement occurred in every month, the period April–September was characterized by larger increases. Given that xylem layers have clearly defined boundaries (see figures in [55]), these months identified the actual growing season.

Data from Site 1, while marred by equipment failures, showed stem growth prior and during the monsoon season, together with the effect of a bark beetle infestation, as described in an earlier article [41]. Data from Site 2 confirmed the spring onset of growth, when mean daily soil temperature measured at the site increased above 4–6 °C (Figure 5). Maximum radial increment occurred between April and June, with a peak in May (the driest month based on average monthly soil moisture; Figure 2), while during the monsoonal rains cumulative SRI increased much more slowly (Figure 5), as incoming solar radiation reached its minimum values for the year (Figure 2; [53]) and soil temperature remained relatively stable or slightly decreased (Figure 5). Monthly growth rates over two consecutive years at Site 2 (Figure 6) showed that non-reversible stem enlargement occurred in every month, even when monthly minimum air temperature was below freezing (Figure 2). In both 2004 and 2005, consecutive months when stem increase exceeded 5% were April through September, with the highest amount of growth taking place in May.

Results from bootstrapped linear correlations between SRI and environmental variables were simplified by only showing significant coefficients (Table 1). Bootstrapped principal component regression coefficients (results not shown) were mostly non-significant, most likely because each monthly multivariate analysis relied on relatively few degrees of freedom (21–31 observations on 12 predictors). Since the sign and relative absolute value of principal component regression coefficients usually matched those derived from simple correlations, we based our interpretation on the latter ones (Table 1). For their brief period of overlap, results from the two sites showed similar responses to environmental changes, except for solar radiation, which played a greater role at Site 2, as shown by positive correlations with SRI at that Site compared with non-significant ones at Site 1. Similarly, negative correlations with precipitation, relative humidity, and soil water (all mutually related) at Site 2 could have been generated by reduced solar radiation because of cloudiness. Soil temperature was important at the beginning of the season (April–May) at both sites. Solar radiation had positive relationships with stem size in the spring (April–May), but negative ones later in the season (June–September), most likely because it increased drought stress by increasing maximum temperature, and was also related to decreased cloudiness, hence lower precipitation. Minimum air temperature was usually positively correlated with radial growth, while maximum air temperature had a positive relationship in spring (April–May), but a negative one in summer (June–September). This latter correlation was likely to be a drought signal, as also shown by the positive correlations with relative humidity and precipitation *vs.* the negative ones with solar radiation (previously mentioned) and vapor pressure deficit at this time of the year. Precipitation had a positive influence on stem size at the start of the monsoon (June–July); later in the season (August–September) a positive correlation also emerged with wind gust and wind speed.

## 4. Discussion

Despite difficulties encountered during the course of the study, accuracy of stem size measurements was minimally affected by the instruments we used. Automated dendrometers use a linear variable differential transformer to transfer stem changes into an electrical signal with a resolution of 4 μm mV^−1^ over a range of 15,000 μm. Thermal expansion and contraction of the dendrometers used in this study was quantified based on their published coefficient of thermal expansion. For point dendrometers, this is 17 μm m^−1^ °C^−1^; since the sensing rod is about 10 cm long, point dendrometers are subject to a 17 μm change for each 10 °C of temperature change, regardless of stem size. Band dendrometers have a smaller coefficient of thermal expansion (11.2 μm m^−1^ °C^−1^), but are subject to large temperature-driven errors when installed on relatively large stems. For instance, when installed on a one-meter stem circumference, a 10 °C temperature change would give an error of 112 μm. There is also the possibility of measurement error due to the thermal expansion and contraction of the tree itself. While there is no standard coefficient of expansion for the trunk of a living tree, published values vary from −3 μm m^−1^ °C^−1^ for Norway spruce to −4 μm m^−1^ °C^−1^ for Scotts pine [25]. These values are smaller than those associated with the sensor themselves, and overall these thermal-driven errors are relatively small, especially when compared to water-related stem changes [54].

Given that annual xylem layers of *Pinus hartwegii* on Nevado de Colima have clearly defined boundaries (see Figures 2 and 3 in [55]), permanent radial expansion outside of April–September was most likely due to moisture storage, which compensated for the dry spring conditions, and allowed for rapid growth before the wet summer monsoon started. Diel stem size variability in woody plants can be caused by tree water status or by formation of new tissues by cell division [56,57]. Maximum insolation in March–May coincides with an earlier arrival of the maximum growth period compared to conifers in northern latitudes [58]. The connection between soil temperature rising above 4–6 °C and the onset of rapid growth agrees with previous studies on high elevation tree species [12].

The spring growing season (April–May) is influenced quite differently than the summer by environmental factors, as one may expect given the June onset of the North American monsoon rains. Before the monsoon, temperature is the main positive signal in radial growth; minimum and maximum soil temperature, as well as maximum air temperature, all directly correlate with stem increment. Solar radiation has a direct positive relationship with stem increment, especially at Site 2, which is on a northern exposure, steeper, rockier, and partially shaded by a nearby rock outcrop. After the rains begin, moisture dominates relationships with stem size, showing a sensitivity to drought that one would not necessarily expect during the wet season. So, for instance, starting in June, radial growth is positively related to precipitation and relative humidity, while it is inversely correlated with maximum air temperature, solar radiation, and vapor pressure deficit. The negative influence of solar radiation is most likely mediated by increased maximum temperature and lack of clouds (hence precipitation). Earlier results on the climatic response of *Pinus hartwegii* tree-ring indices had already indicated June precipitation as the main positive signal [11].

Some limitations on the availability of field measurements throughout the duration of the study were imposed by technological setbacks. Phytograms, *i.e.*, electrodes that are supposed to directly record cambial activity, were installed together with point and band dendrometers, but provided erratic and nonsensical data. As mentioned earlier, band dendrometer readings also had to be discarded because of the excessive influence from bark swelling and shrinking. These automated dendrometer systems had not been previously tested in high elevation environments, and the underground datalogger configuration initially adopted by the manufacturer was particularly sensitive to lightning damage. The aboveground datalogger setup deployed at Site 2 in 2003 proved to be much more resilient, but the manufacturer has now returned to a buried datalogger, albeit of a different design than the original one (William Gensler, Agricultural Electronics Corp., Tucson, AZ; pers. comm., 2009). It is possible, and desirable, that enhanced interest from the scientific community in this type of studies will prompt the development of improved technological tools. Further research should aim at conducting such intensive field monitoring studies for longer periods of time, also in combination with more numerous and more sophisticated electronic instruments (sap flow meters, ground-based NDVI sensors, fiber optic distributed temperature systems, *etc.*).

In conclusion, long-term field-based research remains a fundamental tool for verifying models of eco-hydrologic processes and to document phenologic patterns in ever-changing environments [3]. Understanding how and when trees are growing is also critical to the proper interpretation of dendrochronological records—either from ring width, ring density, or geochemical markers—that extend well beyond the instrumental record. It is therefore suggested that intensive field measurements of stem growth and environmental variables are included wherever tree-ring records are deemed relevant to guide policy making and management decisions.

## Figures and Tables

**Figure 4(a).**
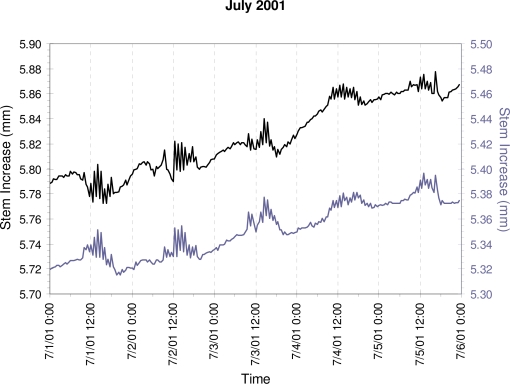

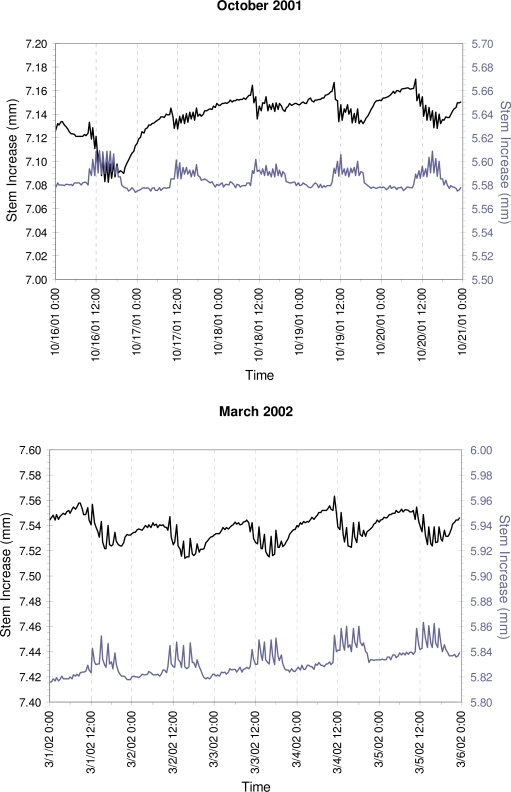
Stem size patterns recorded on two trees at Site 1; line colors (black and gray) identify the same trees in all graphs. Half-hourly measurements during five consecutive days in selected months, with x-axis tick marks at six-hour intervals, and dashed vertical lines marking noon and midnight. Expansion occurs mostly during the night and morning, followed by relatively large oscillations in stem size during the afternoon, and by a more regular contraction during the evening.

**Figure 4(b).**
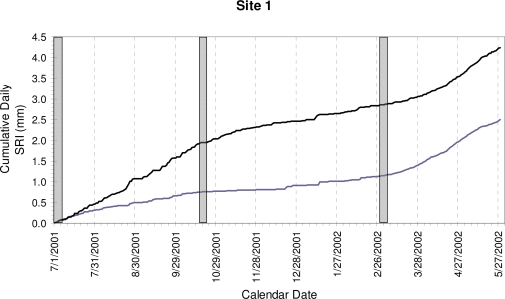
Cumulative Stem Radial Increment (SRI) given by the progressive sum of daily SRI, which was computed from the half-hourly measurements by subtracting the maximum stem size during one 24-hour period from the maximum stem size in the following 24-hour period. Gray-shaded rectangles indicate the days plotted in (a).

**Table 1.** Bootstrapped linear correlations between daily stem radial increment (SRI; S1 = Dendrometer Site 1; S2 = Dendrometer Site 2) and daily environmental variables ^(1)^ measured by the automated weather station during the April-September growing season. Only coefficients with confidence level >95% for months that had at least 21 days of available observations are shown ^(2)^.

**Month**	**SolRad**		**Prec**		**TSmax**		**TSmin**		**TAmax**		**TAmin**		**RH**		**SWV**		**AirPr**		**WG**		**WS**		**VPD**	
	**S1**	**S2**	**S1**	**S2**	**S1**	**S2**	**S1**	**S2**	**S1**	**S2**	**S1**	**S2**	**S1**	**S2**	**S1**	**S2**	**S1**	**S2**	**S1**	**S2**	**S1**	**S2**	**S1**	**S2**
Jul 2001	ns	/	ns	/	ns	/	ns	/	ns	/	ns	/	0.54	/	ns	/	−0.42	/	ns	/	ns	/	−0.50	/
Aug 2001	−0.41	/	ns	/	ns	/	ns	/	−0.44	/	0.41	/	0.37	/	ns	/	ns	/	0.48	/	ns	/	−0.36	/
Sep 2001	−0.48	/	0.45	/	ns	/	ns	/	−0.59	/	0.47	/	0.51	/	ns	/	−0.51	/	0.55	/	0.43	/	−0.51	/

Apr 2002	ns	0.39	ns	Ns	0.46	0.57	0.54	0.55	0.35	0.58	0.44	0.43	ns	ns	−0.45	−0.54	/	/	ns	ns	ns	ns	/	/
May 2002	ns	0.64	ns	−0.40	0.46	0.55	0.60	0.35	0.31	0.58	0.46	0.63	ns	−0.52	−0.29	ns	/	/	ns	ns	ns	0.45	/	/
Jun 2002	−0.54	/	0.54	/	ns	/	ns	/	−0.47	/	ns	/	0.74	/	ns	/	ns	/	ns	/	ns	/	−0.72	/
Jul 2002	−0.56	/	0.41	/	ns	/	0.49	/	ns	/	0.35	/	ns	/	ns	/	ns	/	−0.31	/	−0.37	/	ns	/
Aug 2002	−0.49	/	ns	/	ns	/	0.45	/	−0.45	/	0.41	/	ns	/	ns	/	−0.53	/	0.46	/	0.47	/	ns	/
Sep 2002	−0.47	/	0.48	/	ns	/	ns	/	−0.61	/	0.38	/	ns	/	ns	/	−0.46	/	0.57	/	0.52	/	ns	/

Jun 2003	−0.56	−0.43	0.41	Ns	ns	ns	ns	ns	ns	ns	0.36	ns	0.43	ns	/	/	ns	−0.38	ns	ns	ns	ns	−0.42	ns
Jul 2003	−0.59	−0.46	0.63	0.32	ns	ns	ns	ns	−0.47	ns	ns	ns	ns	ns	/	/	ns	−0.31	ns	ns	ns	ns	ns	ns
Aug 2003	−0.49	−0.47	ns	Ns	ns	ns	0.38	ns	−0.47	−0.44	0.51	0.38	ns	ns	/	/	ns	ns	0.45	0.35	0.54	0.40	ns	ns
Sep 2003	−0.45	−0.50	ns	Ns	ns	−0.34	ns	ns	−0.60	−0.72	0.41	0.31	0.61	0.59	/	/	ns	−0.36	ns	ns	0.37	0.46	−0.57	−0.56

Apr 2004	/	0.60	/	−0.29	/	0.72	/	0.70	/	0.54	/	0.67	/	−0.53	/	−0.68	/	ns	/	ns	/	ns	/	0.69
May 2004	/	0.33	/	Ns	/	0.56	/	0.43	/	0.61	/	0.46	/	−0.42	/	ns	/	ns	/	ns	/	ns	/	0.52
Jun 2004	/	−0.57	/	0.57	/	ns	/	ns	/	−0.53	/	0.53	/	0.47	/	ns	/	ns	/	0.50	/	0.49	/	−0.45
Jul 2004	/	Ns	/	0.43	/	ns	/	ns	/	−0.40	/	ns	/	0.33	/	ns	/	ns	/	ns	/	ns	/	−0.32
Aug 2004	/	Ns	/	Ns	/	ns	/	ns	/	ns	/	ns	/	ns	/	ns	/	ns	/	ns	/	ns	/	ns
Sep 2004	/	Ns	/	Ns	/	ns	/	ns	/	ns	/	ns	/	ns	/	ns	/	ns	/	ns	/	ns	/	ns

Apr 2005	/	Ns	/	Ns	/	0.58	/	0.52	/	0.43	/	0.35	/	−0.38	/	−0.64	/	ns	/	ns	/	ns	/	0.44
May 2005	/	Ns	/	Ns	/	ns	/	ns	/	ns	/	ns	/	−0.39	/	ns	/	ns	/	ns	/	ns	/	0.41
Jun 2005	/	Ns	/	Ns	/	ns	/	ns	/	ns	/	ns	/	ns	/	ns	/	ns	/	ns	/	ns	/	ns
Jul 2005	/	−0.43	/	0.44	/	ns	/	ns	/	−0.54	/	ns	/	0.54	/	0.51	/	ns	/	ns	/	0.41	/	−0.53
Aug 2005	/	−0.49	/	Ns	/	ns	/	ns	/	−0.58	/	ns	/	0.56	/	0.41	/	ns	/	ns	/	0.37	/	−0.63
Sep 2005	/	−0.34	/	0.57	/	ns	/	ns	/	−0.41	/	ns	/	0.45	/	ns	/	ns	/	0.35	/	0.43	/	−0.43

(1) SolRad = total incoming solar radiation (MJ m^−2^); Prec = total precipitation (mm); TSmax, TSmin = soil temperature (°C), respectively maximum and minimum; TAmax, TAmin = air temperature (°C), respectively maximum and minimum; RH = mean relative humidity (%); SWV = mean soil moisture content (%); AirPr = mean barometric pressure (hPa); WG = maximum wind gust (km hr^−1^); WS = mean wind speed (km hr^−1^); VPD = mean vapor pressure deficit (hPa).

(2) / = data not available; ns = correlation not significant (confidence level ≤ 95 %).
